# *Olax scandens* Mediated Biogenic Synthesis of Ag-Cu Nanocomposites: Potential Against Inhibition of Drug-Resistant Microbes

**DOI:** 10.3389/fchem.2020.00103

**Published:** 2020-02-28

**Authors:** Anzar Abdul Mujeeb, Nuha Abeer Khan, Fauzia Jamal, Khan Farheen Badre Alam, Haris Saeed, Shadab Kazmi, Ansam Wadia Faid Alshameri, Mohammad Kashif, Irfan Ghazi, Mohammad Owais

**Affiliations:** ^1^Interdisciplinary Biotechnology Unit, Aligarh Muslim University, Aligarh, India; ^2^Plant Molecular Biology and Genetic Engineering Division, The National Botanical Research Institute, Council of Scientific and Industrial Research, Lucknow, India; ^3^Department of Biochemistry, School of Life Sciences, University of Hyderabad, Hyderabad, India

**Keywords:** biogenic, Ag-Cu NCs, anti-biofilm potential, reactive oxygen species (ROS), antimicrobial potential

## Abstract

In the present study, we have synthesized silver-copper nanocomposites (Ag-Cu NCs) using an *Olax scandens* leaf extract (green synthesis method) and evaluated their antimicrobial potential against less susceptible pathogens. The kinetics of Ag-Cu NCs synthesis was followed by UV-VIS and fluorescence spectroscopy. The physicochemical characterization of as-synthesized Ag-Cu NCs was executed using electron microscopy, Energy Dispersive X-Ray, Fourier Transform Infrared Spectroscopy, and a Differential Light Scattering method. As-synthesized Ag-Cu NCs induced the formation of Reactive Oxygen Species (ROS), thereby causing alteration and decrementation of cellular proteins, DNA, lipids, etc., and eventually leading to cell death, as determined by a Live/Dead assay. Next, we assessed the anti-biofilm potential of as-synthesized Ag-Cu NCs against biofilm forming bacteria. The as-synthesized Ag-Cu NCs, when compared to monometallic silver nanoparticles, exhibited significantly higher anti-microbial activity against both sensitive as well as drug resistant microbial isolates.

## Introduction

Metallic nanoparticles have found diversified applications in the area of electronics, food, and, most profoundly, several biomedical-related fields, especially for their antimicrobial potential and also for their use as a diagnostic tool (Klebowski et al., 2018; Kumar et al., 2018; Azharuddin et al., 2019; Moutsiopoulou et al., 2019). The nano-sized metal-based particles have also been considered as promising tools for targeted drug delivery (Patra et al., 2018), imaging (Chen et al., 2018), sensors (Colino et al., 2018), synthetic inhibitors (Ma et al., 2018), etc. Nanomaterials have strikingly unique physicochemical properties (Ahmed et al., 2017), such as large surface area to mass ratio, ultra-small size, and high reactivity, which impart unusual mechanical (Guo et al., 2013), optical (Jackson and Halas, 2001), electrical, and chemical properties to as-formed particles.

In the medical field, nanosized metal particles with modified physical and chemical attributes have been utilized excessively. Plant or microbial extract-based fabrication of metal nanoparticles has been exploited in the recent past to revolutionize the field of nanotechnology (Yang et al., 2011). Among various metal-based nanostructures, silver nanoparticles have been reported to show characteristic physico-chemical properties with superior clinical and therapeutic properties—more so than compared to its parent salt. Similarly, copper nanoparticles have also been reported to show high thermal stability, electrical conductivity, optical, catalytic, antimicrobial properties (Lee and Jun, 2019), etc.

There is growing trend of employing nanocomposites as a substitute for single metal-based nanoparticles. Nanocomposites, as the name indicates, are basically combinations of more than one core material and show better attributes as compared to single metal-based nanoparticles. New approaches have been exploited to design nanocomposites that exhibit more versatile structures and features as compared to the original contributing monometallic counterparts (He et al., 2018).

Bio mediated synthesis of nanocomposites is on the rise due to the growing need to develop environmentally benign technologies. Interestingly, nanocomposites comprising of a silver and copper combination show improved physical and chemical properties as compared to the individual parent nanoparticles (Peszke et al., 2017).

There was a great deal of concern regarding protocols employed in the fabrication of metal-based nanoparticles since they require high temperature, pressure, and specific reaction conditions. The chemical synthesis approach was not only tedious and expensive; it also poses a great deal of health hazards as well. The introduction of bio-mediated synthesis of the metal nanoparticles has revolutionized the overall nanoparticle fabrication process. Besides making the whole synthesis process very simple and cost effective, the employed green synthesis approach also circumvents environmental and health-related issues (Singh et al., 2018).

In the present study, we have synthesized silver-copper nanocomposites using a simple, cheap, and environment friendly biological synthesis method (Thakkar et al., 2010). *Olax scandens* leaf extract was used as a reducing and capping agent for the bio-mediated conversion of silver and copper salts to a nanocomposite structure. *Olax* is a medicinally (Khare, 2007) important plant found throughout tropical India. It contains various phytochemicals, such as oleanolic acid, and β-sitosterol, etc. (Mukherjee et al., 2013). Previous studies have shown that the leaf extract of the *Olax* contains phytochemicals along with a 12–17 KDa proteins, which exhibit strong fluorescence upon their internalization by living cells (Patra et al., 2014).

The antimicrobial effect of the nanocomposites was established by determining its antimicrobial potential against some common bacterial and fungal microbes, viz., *E. coli, S. aureus, P. aeruginosa, K. pneumoniae, C. albicans*, F. *moniliforme*, etc.

## Experimental Procedures

### Materials

Silver nitrate (AgNO_3_) and Copper Sulfate (CuSO_4_.5H_2_O) were purchased from Merck research laboratories private limited (India). Luria Broth (LB), Brain Heart Infusion (BHI), Yeast Extract Peptone Dextrose (YEPD), and agar powder were procured from Hi-media (India). FITC, SYTO9, and PI dye for fluorescence microscopy were purchased from Sigma-Aldrich (St. Louis, MO). The standard bacterial and fungal strains, which were obtained from JNMCH, AMU, and Aligarh, were sub-cultured in Luria Broth and Brain Heart Infusion. The cultures were stored at −20°C as 20% glycerol stock for long-term preservation. All experiments were performed with log phase culture isolates.

### Preparation of *Olax scandens* Leaf Extract

The plant *Olax scandens* belongs to the family Olacaceae and is commonly known as parrot O*lax*. The plant is a shrubby climber in nature, and it generally grows in tropical countries, including India. *Olax scandens* leaves were thoroughly washed with distilled water three times. The leaves were allowed to dry in shade and were subsequently crushed to get a fine powder. A known amount (20 gm) of the dried plant leaves was suspended in 100 ml deionized water and boiled. The extract was filtered and finally stored at −20°C until further use.

### Biosynthesis of Silver and Copper Nanoparticles

Silver and copper nanoparticles were synthesized using standard published protocols (Ashraf et al., 2016). *Olax scandens* leaf extract was used as a reducing (initiator) and stabilizing/capping agent to induce the formation of metallic nanoparticles.

For the synthesis of silver nanoparticles, 2 mL of *Olax scandens* leaf extract stock was mixed with 1.75 mL of silver nitrate solution (20 mM), thus keeping the final reaction volume 5 mL. The reaction mixture was stirred on a shaker for 24 h at room temperature (25°C). A change in color (Kelly and Johnston, 2011) from light yellow to black was observed to accomplish NP synthesis.

In case of copper nanoparticles, 2 mL of *Olax scandens* leaf extract was added in 20 mM copper sulfate (CuSO_4_.5H_2_O) solution. A known volume (100 μL) of NaOH solution was also added to keep the incubation condition alkaline (Mohindru and Garg, 2017). The final volume of the solution was maintained up to 5 mL with water. The mixture was kept at room temperature on a shaker for 24 h and a change in color from yellow to green was observed as a feature to ascertain nanoparticle formation.

### Bio-Mediated Synthesis of Silver-Copper Nanocomposites (Ag-Cu NCs)

*Olax scandens* leaf extract was used as a reducing and stabilizing/capping agent for nanocomposite fabrication. The color, shape, size, and stability of the nanocomposites depends on the concentration/volume of the reducing agent used. A stock solution at the strength of 20 mM for both AgNO_3_ and CuSO_4_.5H_2_O was prepared by dissolving respective salts in deionized water. Next, 500 μL of *Olax scandens* leaf extract was added to a mixture of 250 μL of AgNO_3_ and 250 μL of CuSO_4_.5H_2_O (total reactant volume of 5.0 mL). The sample was incubated at room temperature for 24 h on a shaker. The color of the solution changed from light yellow to brown indicating the formation of Ag-Cu Nanocomposite (Ag-Cu NCs).

### Characterization of Ag-Cu NCs

#### UV-Visible and Fluorescence Spectroscopy

Ultraviolet-Visible (UV) spectrum of as-prepared Ag-Cu NCs was recorded on a double beam spectrophotometer (Shimadzu) operated at a resolution of 1 nm in the range of 300–700 nm. The fluorescence spectrum of as-synthesized Ag-Cu NCs was recorded using an excitation wavelength of 320 nm at room temperature. The emission spectrum was recorded in the range of 400–550 nm.

#### Electron Microscopic Studies

The size dimensions as well as surface morphology of the as-synthesized nanocomposites were characterized by TEM and SEM techniques. A sample was prepared by placing a drop of reactant product over a 200-mesh copper grid that was then covered by the carbon-stabilized formvar film used for probing the as-synthesized AgNPs, CuNPs, and Ag-Cu NCs. Uranyl acetate (2%w/v) was used as a negative stain. The excess fluid from the sample was removed before TEM analysis. TEM study was performed on JEOL model electron microscope. For surface morphological analysis of the as-synthesized Ag-Cu NCs, scanning electron microscopy (SEM) was performed using JSM67500F, a JEOL model. Elemental composition of the as-synthesized nanocomposite was determined using Energy Dispersive X-Ray Analyzer equipped with Scanning Electron Microscope (SEM-EDX).

#### Dynamic Light Scattering (DLS)

The size analysis of the as-synthesized nanocomposites was executed using Dynamic Light Scattering (DLS, using Dynopro-Tc-04 instrument; Protein Solution, Wyatt Technology, Santa Barbara, CA), which measures diffusion of dispersed particle. The average hydrodynamic diameter was calculated using the Stokes-Einstein equation. The scattered light intensity was detected at 90° of the incident beam. Briefly, PB buffer [PB pH 7.4] was used to re-suspend the as-synthesized NPs and NCs. The solution obtained after passing through 0.22 μm filter (Millipore) was subjected to various size determining measurements. The data was analyzed in the default mode. The average value of 20 runs (done in triplicate) was considered for assessment of the size of the as-synthesized Ag-Cu NCs.

#### FTIR Spectroscopy

In order to identify the presence of various functional groups associated with the as-synthesized nanocomposite, it was analyzed by Flourier Transform Infrared (FTIR) Spectroscopy. An as-synthesized NPs and NCs sample disc was co-prepared along with Kerr crystals as a beam splitter.

### Determination of MIC of As-Synthesized Ag-Cu NCs Against Various Bacterial and Fungal Pathogens

MIC is considered to be a significant parameter that help to ascertain the sensitivity of a microorganism to a particular antimicrobial agent. The MIC value of the as-synthesized Ag-Cu NCs was determined by a micro-dilution method against various bacterial and fungal strains as recommended by NCCLS. Various clinical isolates used in the study (viz., *E. coli, S. aureus, P. aeruginosa, K. pneumoniae, C. albicans*, and F. *moniliforme*) were gifted by Prof M. Shahid, Department of Microbiology, JNMCH, AMU, Aligarh. The MIC value was estimated on the basis of viability test performed in 96-well micro-dilution plates.

### Antimicrobial Potential of Ag-Cu NCs as Determined by the Agar Disc Diffusion Method

The agar disc diffusion technique was also used to evaluate antimicrobial activity of as-synthesized Ag-Cu NCs. An aliquot (100 μL) of the suspended culture was spread evenly on the petri-plate using a sterile plastic spreader and incubated at 37°C. After the 1-h incubation period, wells were bored on the agar plate. Subsequently, an increasing amount of as-synthesized Ag-Cu NCs (1 mg/ml stock solution) or standard antibiotic was dispensed into the various wells in a given agar plate. Combinations of nanocomposites and antibiotics were also tested for their antimicrobial potential. Zone of inhibition was determined by measuring the clear region around each well corresponding to Ag-Cu NCs mediated bacterial clearance after 24 h. The same protocol was used to determine antifungal potential of as-synthesized Ag-Cu NCs against *C. albicans* and *F. moniliforme*.

### Fluorescence Microscopic Study Based on Live/Dead Assay to Assess the Antimicrobial Activity of Ag-Cu NCs Against Microbial Pathogens

A fluorescence microscopic study was performed to elucidate antimicrobial activity of Ag-Cu NCs. Ag-Cu NCs (at MIC value) was co-incubated with the overnight grown bacterial culture at 37°C for 3 h in the dark. Bacterial cells were harvested by centrifugation of cultured bacteria at 2,500 × g for 5 min. The pellet was re-suspended in 1 ml PBS buffer. The cells were stained by incubation with 10 μL of PI stock to achieve a final concentration of 1 μg/ml. The solution was incubated for 3 h at 37°C. Likewise, a control bacterial cell culture (no Ag-Cu NCs) was grown under similar conditions and subsequently stained with SYTO9 dye. A drop of the above-prepared sample was placed on a glass slide and mounted with a cover slip. The treated cells were examined under a fluorescence microscope (40×).

### Biofilm Inhibition Potential of As-Synthesized Ag-Cu NCs

*S. aureus* was cultured in Luria Bertani broth medium at 37°C overnight. An aliquot of log phase bacterial culture (having 10^6^ cfu/ml) was suspended in broth (500 μL) and dispensed on the sterile cover slip. The setup was left for 24 h at 37°C for the growth of biofilm. Mature biofilm was washed and incubated with 100 μL Ag-Cu NCs (from 1 mg/ml stock solution). After a stipulated time period, coverslips were fixed in 4% PFA followed by washing and cells were then stained with FITC dye. Stained coverslips were then washed thrice with sterile PBS and analyzed under the Zeiss, Axiocam Imager MRM M2 fluorescence microscope. Both the treated and control samples were observed under a fluorescence microscope (40× magnification).

### Intracellular ROS Production by As-Synthesized Ag-Cu NCs

Metal nanoparticles induced intracellular ROS production to kill the target bacterial/fungal cells. The ROS production was measured by employing fluorescent probe 2,7-dichlorofluorescein diacetate (DCHF-DA). The DCHF-DA passively diffuses through the cell membrane, and, once internalized, it gets deacetylated by intracellular esterases to form a non-fluorescent 2,7-dichloroflour-escein (DCHF). The DCHF reacts with the generated ROS to produce the fluorescent product 2,7-dichlorofluorescein (DCF) which gets trapped within the cell making it fluorescent. Briefly, the log phase bacteria (10^6^ Cfu/ml) were washed three times with fresh medium. DCHF-DA was mixed with the bacterial culture and incubated in shaking incubator for 30 min at 37°C. The cells were pelleted down by centrifugation and washed to remove the unbound DCHF. The treated bacterial cells were visualized under a fluorescence microscope (Zeiss model, USA) for validation of Ag-Cu NCs induced ROS generation. The higher fluorescence intensity corresponds to the elevated ROS production.

## Results

### UV-Visible Spectroscopy

**Figure 1:**
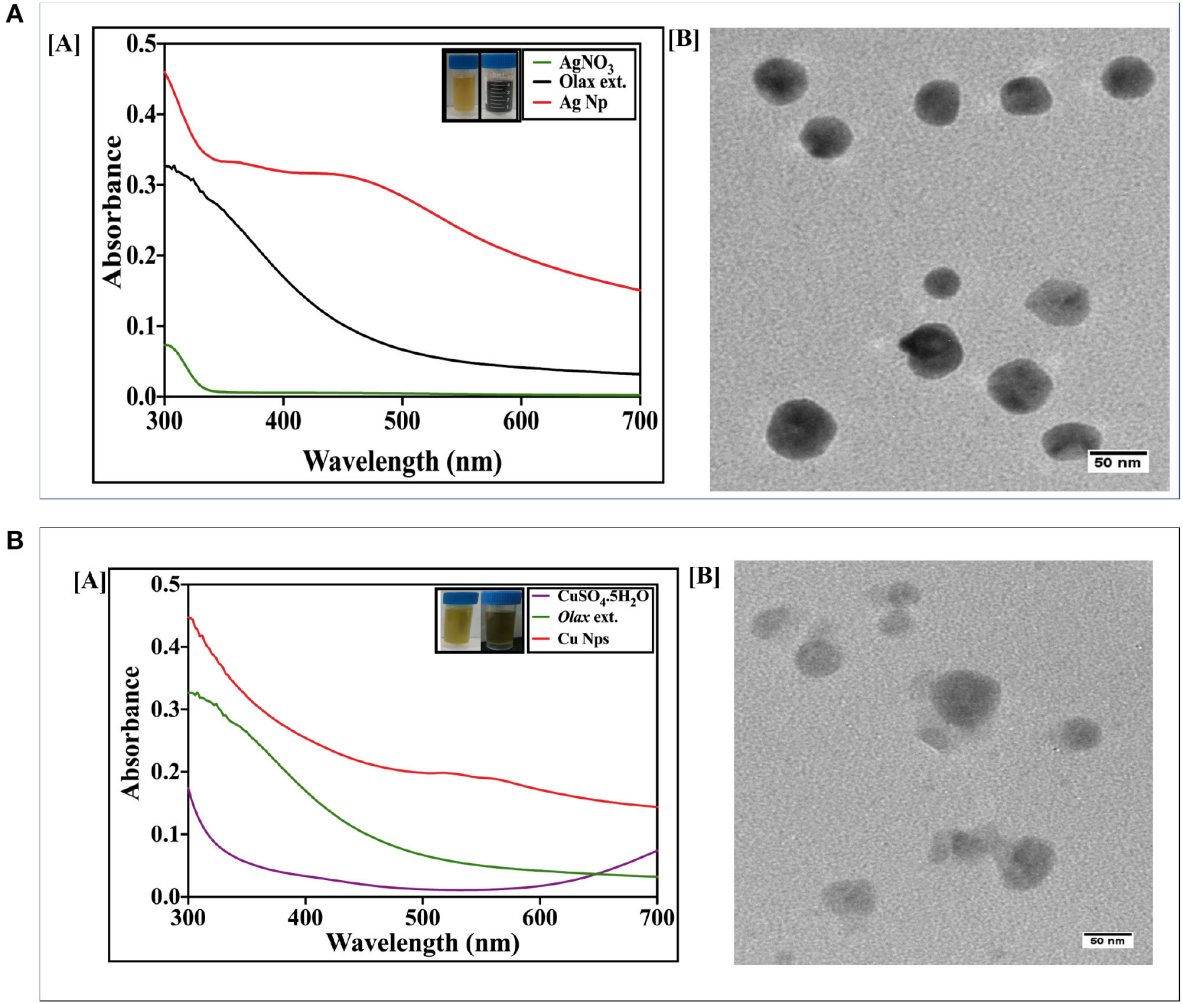
**(A)** [A] UV-Visible absorption spectrum of as-synthesized AgNPs fabricated employing *Olax* leaf extract: UV-Visible spectra of silver nanoparticles formed after incubation of AgNO_3_ (20 mM) with *Olax scandens* leaf extract. The characteristic peak of AgNPs was observed at around 480 nm, thus signifying the formation of silver nanoparticles. [B] TEM analysis depicting shape and size of as-synthesized AgNPs. Representative TEM images of silver nanoparticles synthesized using *Olax scandens* leaf extract showing simultaneous presence of ovoid and hexagonal shaped structures. Inset shows change in color as function of AgNPs synthesis. **(B)** [A] UV-visible absorption spectrum of as-synthesized Copper nanoparticles fabricated using *Olax* leaf extract: [A] UV-Visible spectra of copper nanoparticles formed after incubation of CuSO_4_.5H_2_O (20 mM) with *Olax scandens* leaf extract. The characteristic peak at 560 nm was observed in case of copper nanoparticles. [B] TEM analysis depicting shape and size of as-synthesized CuNPs. Representative TEM images of copper nanoparticles synthesized using extract of *Olax scandens* leaf showing simultaneous presence of varying shaped nano-sized structures. Inset shows that the fabrication of CuNPs is accompanied with change in color.

In general, incident electromagnetic radiation stimulates resonant oscillation of conduction band electrons between positive and negative permittivity material. The localized oscillation of the electrons ensues in surface plasmon resonance to the metal nanoparticles that can be exploited to characterize as-formed nanoparticles. The bio-mediated reduction of silver and copper ions to their respective nanoparticles was monitored by UV-VIS spectroscopy. Initially, we established the potential of *Olax scandens* leaf extract to mediate synthesis of monometallic silver and copper nanoparticles. While the silver nitrate solution failed to absorb at 480 nm, there was a characteristic spectrum with a prominent peak at 480 nm in the case of Ag the nanoparticles (Figure 1A). We observed a change in color (from light yellow to black) in the reaction mixture upon treatment of AgNO_3_ with *Olax scandens* leaf extract. The color change can be correlated with the reduction of silver ions (Ag^+^) to silver nanoparticles.

The synthesis of copper nanoparticles was also followed in the similar way, wherein, the copper ions got reduced when the copper sulfate (CuSO_4_.5H_2_O) solution was mixed with the plant extract and incubated on a shaker for 24 h. The progress of the reaction was followed by monitoring change in color of the reactants. The light yellow color of the CuSO_4_ solution turned dark green in time-dependent manner. The absorption spectra showed a peak at 560 nm corresponding to copper nanoparticles (Figure 1B).

**Figure 2:**
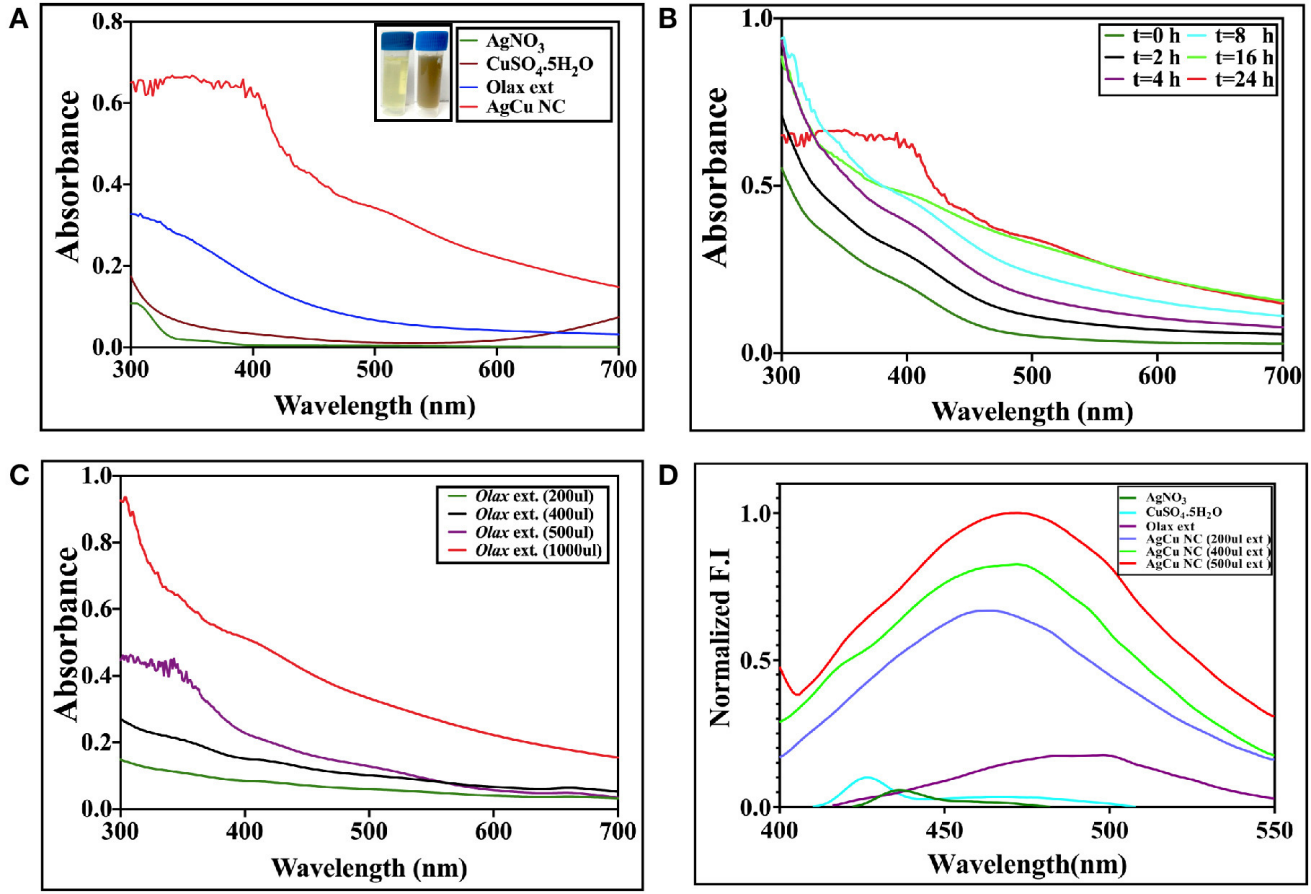
**(A)** UV-visible absorption spectrum of as-synthesized Ag-Cu nanocomposite synthesized using *Olax* leaf extract: UV-Visible spectra of Ag-Cu nanocomposite formed after incubation of equimolar solution of AgNO_3_ (20 mM) and CuSO_4_.5H_2_O (20 mM) with *Olax scandens* leaf extract. **(B)** Time-dependent kinetics of Ag-Cu nanocomposite synthesis in the presence of *Olax scandens* extract: to elucidate time-dependent kinetics of Ag-Cu nanocomposite synthesis, the incubation mixture was scanned spectrophotometrically (UV-VIS) at various time points (0–24 h). The characteristic surface plasmon resonance (SPR) band of the Ag-Cu nanocomposites progressively increased in a time-dependent manner, signifying the formation of the Ag-Cu nanocomposites. **(C)** The characteristic peak of the SPR band corresponding to the Ag-Cu nanocomposites progressively shifted toward a higher wavelength with amplification in intensity when incubated with increasing amounts of O*lax scandens* leaf extract. **(D)** Normalized fluorescence spectra of the as-synthesized Ag-Cu NCs: Fluorescence spectra of as-synthesized Ag-Cu NCs was recorded in the range of 400–550 nm; an excitation wavelength of 320 nm was used. As evident from the figure, an increase in concentration of *Olax* leaf extract resulted in enhancement of the fluorescence intensity.

Next, silver-copper nanocomposites (Ag-Cu NCs) were synthesized by incubation of silver nitrate and copper sulfate-equimolar concentration solution, with *Olax scandens* leaf extract. The mixture was incubated for 24 h. The NC synthesis involved a change in color as the solution turned from light yellow to brown. The UV-VIS absorption spectra of as-synthesized NCs demonstrated a surface plasmon resonance at about 520 nm (Figure 2A). The characteristic peak was midway between the peaks corresponding to pure Ag and Cu nanoparticles, i.e., at 480 and 560 nm, respectively.

### Kinetics of Ag-Cu NCs Synthesis Upon Co-incubation of a Mixture of Copper Sulfate and Silver Nitrate Solution With *Olax* Leaf Extract

The incubation of two metal salts with *Olax scandens* leaf extract resulted in a characteristic peak at 520 nm. UV-VIS spectra showed an initial peak around 520 nm in the as-synthesized Ag-Cu NCs (Figure 2A). The time kinetics study was followed for extended time period ranging from 0 to 24 h (Figure 2B). No further change in the intensity of characteristic peaks in the spectrum (after 24 h) is suggestive of the completion of Ag-Cu NCs formation (data not shown).

The effect of increasing concentrations of leaf extract on bio mediated synthesis of Ag-Cu NCs was also examined (Figure 2C). An increment in *Olax* leaf extract content ensued in increase in the absorption spectra peak. This can be correlated with more profound synthesis of nanocomposites due to the increasing presence of reducing and capping agents in the extract.

### Fluorescence Spectroscopy

The fluorescence spectrum of as-synthesized Ag-Cu NCs was plotted using an excitation wavelength of 320 nm. The emission was recorded from 400 to 550 nm. Ag-Cu NCs showed a fluorescence peak at 450 nm (Figure 2D). The intensity of characteristic fluorescence spectrum increased with increment in the amount of leaf extract used in bio-mediated synthesis of as-formed NCs.

### Electron Microscopy

**Figure 3:**
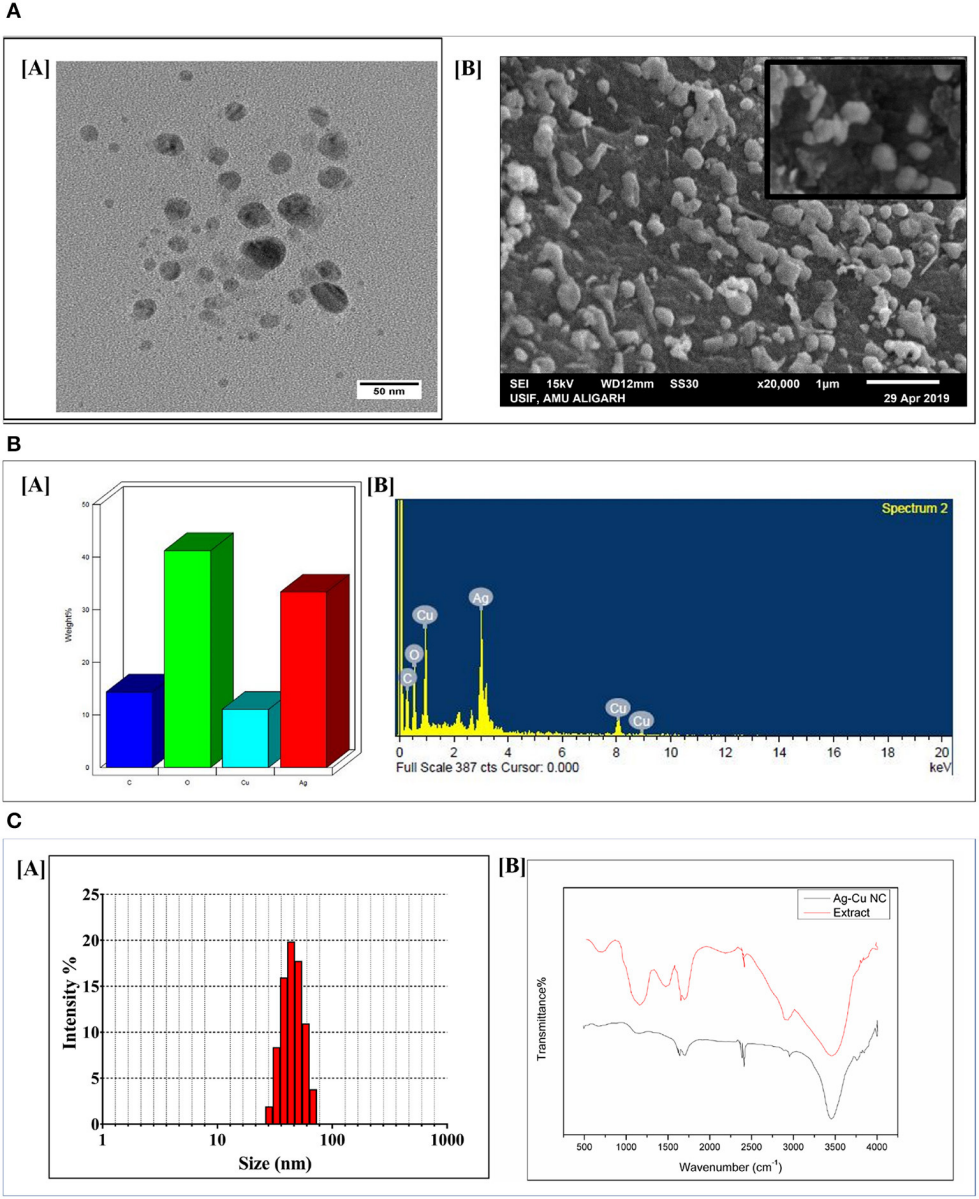
**(A)** [A] TEM/SEM analysis depicting shape and size of as-synthesized Ag-Cu nanocomposites: representative TEM images of as-synthesized Ag-Cu nanocomposites synthesized using *Olax scandens* leaf extract. Ag-Cu nanocomposites with varying shapes and sizes had been observed. [B] SEM analysis of the as-synthesized Ag-Cu nanocomposites using *Olax scandens* leaf extract. Inset shows the HR-SEM at higher magnification. **(B)** [A] Determination of elemental composition of as-synthesized Ag-Cu nanocomposites: representative bar graphs corresponding to the elemental composition of the as-synthesized nanocomposites were determined using Energy Dispersive X-Ray Analyzer equipped with Scanning Electron Microscope (SEM-EDX). **(B)** Graph showing the spectral composition of the composition of Ag-Cu NCs. The percentages of Ag, Cu, C, and O in the as-synthesized Ag-Cu NCs were found to be 33.35, 11.05, 14.37, and 41.22%, respectively. **(C)** [A] Size analysis of the as-synthesized Ag-Cu nanocomposites as determined by DLS analysis. Dynamic Light Scattering (DLS) particle size analysis suggested overall particle radii of the as-synthesized Ag-Cu nanocomposites to be approximately in the range 20–50 nm. [B] FTIR spectrum of as-synthesized Ag-Cu nanocomposites: FTIR spectra of as-synthesized Ag-Cu nanocomposites generated after incubation of *Olax scandens* leaf extract with aqueous solution of a mixture of AgNO_3_ andCuSO_4_.5H_2_O. The red curve represents pure *Olax scandens* leaf extract, and the black curve corresponds to as-synthesized Ag-Cu nanocomposites.

The shape and the size of the as-synthesized Ag-Cu NCs was determined by SEM and TEM analysis. The size of Ag-Cu NCs was found to be in the range of 10–20 nm via TEM micrograph at 100,000× (Figure 3A). The SEM image revealed spherical shape and uniform size distribution of the as-synthesized nanocomposites (Figure 3A). Further EDX spectra (Figure 3B) ascertained the presence of Ag, Cu, C, and O in the as-synthesized Ag-Cu NCs to be 33.35, 11.05, 14.37, and 41.22%, respectively. This analysis additionally confirms the absence of any other moiety in the prepared nanocomposites.

### Dynamic Light Scattering (DLS)

The size of the as-synthesized nanocomposites as observed by Dynamic Light Scattering analysis was found to be in the range from 20 to 50 nm (Figure 3C). In general, DLS size measurements are larger as compared to the size dimensions determined by TEM analysis. TEM analysis provides information about the size and shape of individual nanoparticles dried under high vacuum, whereas Dynamic Light Scattering (DLS) measures particle dimensions upon their dispersion in a given medium. The size corresponds to average hydrodynamic diameter of the particle that is determined by Strokes-Einstein equation. This generally ensues in discrepancies between TEM- and DLS-based size determination as the former corresponds to dried form while hydrodynamic and electro kinetic parameters are operative in DLS measurements.

### Identification of Functional Groups Present in Ag-Cu NCs Employing FTIR Spectroscopy

In order to characterize the functional groups, present in the Ag-Cu NCs, a Fourier Transform Infrared (FTIR) Spectroscopy was conducted (Figure 3C). The presence of various chemical groups gave rise to specific peaks corresponding to various wave numbers. We found N-H stretching vibrations of amines or amide linkages (strong peaks at 3,100–3,680 cm^−1^), amide bands of proteins (at 1,634 cm^−1^ and 1,448 cm^−1^), C-N stretching vibrations of aromatic and aliphatic amines (the bands observed at 1,335 and 1,264 cm^−1^, respectively), and C-O stretching vibrations related to carboxylate and alcoholic groups (weaker bands at 998 cm^−1^) on the surface of Ag-Cu NCs. It can be concluded that the nanocomposites were coated by proteins present in the plant extract. The biomolecules of extract may get adsorbed on the surface of NCs and impart them stability, thereby preventing their aggregation.

### Anti-microbial Activity of Ag-Cu NCs

The antimicrobial action of Ag-Cu NCs was evaluated against both Gram-negative and Gram-positive bacteria. We also established antifungal activity of as-synthesized Ag-Cu NCs against *F. moniliforme* and *C. albicans*. Nanocomposites were found to be more effective against both *E. coli* and *S. aureus* as compared to silver and copper based plain nanoparticles (Table 1). Next, the anti-microbial efficacy of silver-copper nanocomposites was determined against various strains of bacteria and fungi, e.g., *K. pneumoniae, P. aeruginosa, F. moniliforme*, and *C. albicans* (Table 2).

**Table 1:** Comparison of MIC values of nanoparticles and nanocomposites.

**NPs and NC**	***E. coli***	***S. aureus***
Ag np's	125 μg/ml	250 μg/ml
Cu np's	250 μg/ml	250 μg/ml
AgCu NC	62 μg/ml	125 μg/ml

**Table 2:** MIC values of Ag-Cu-NCs against bacterial and fungal strains.

**Microbes**	
*E. coli*	62 μg/ml
*S. aureus*	125 μg/ml
*K. pneumoniae*	125 μg/ml
*P. aeruginosa*	125 μg/ml
*F. moniliforme*	250 μg/ml
*C. albicans*	125 μg/ml

### Minimum Inhibitory Concentration (MIC) of Various As-Synthesized NPs/NCs

We also determined MIC of various as-synthesized nanosized metal particles. The MIC value of plain AgNPs was found out to 125 μg/ml against *E. coli* and 250 μg/ml for *S. aureus*. The CuNPs with MIC value of 250 μg/ml against both *E. coli* and *S. aureus* was less efficient as compared to the AgNPs. There was a dramatic increase in the antimicrobial properties of the as-synthesized Ag-Cu NCs as it exhibited MIC value of 62.5 μg/ml against *E. coli*. The MIC value of Ag-Cu NCs was 125 μg/ml for *S. aureus* (Table 1).

### Antibacterial Potential of Ag-Cu NCs as Determined by Agar Disc Diffusion Assay

**Figure 4:**
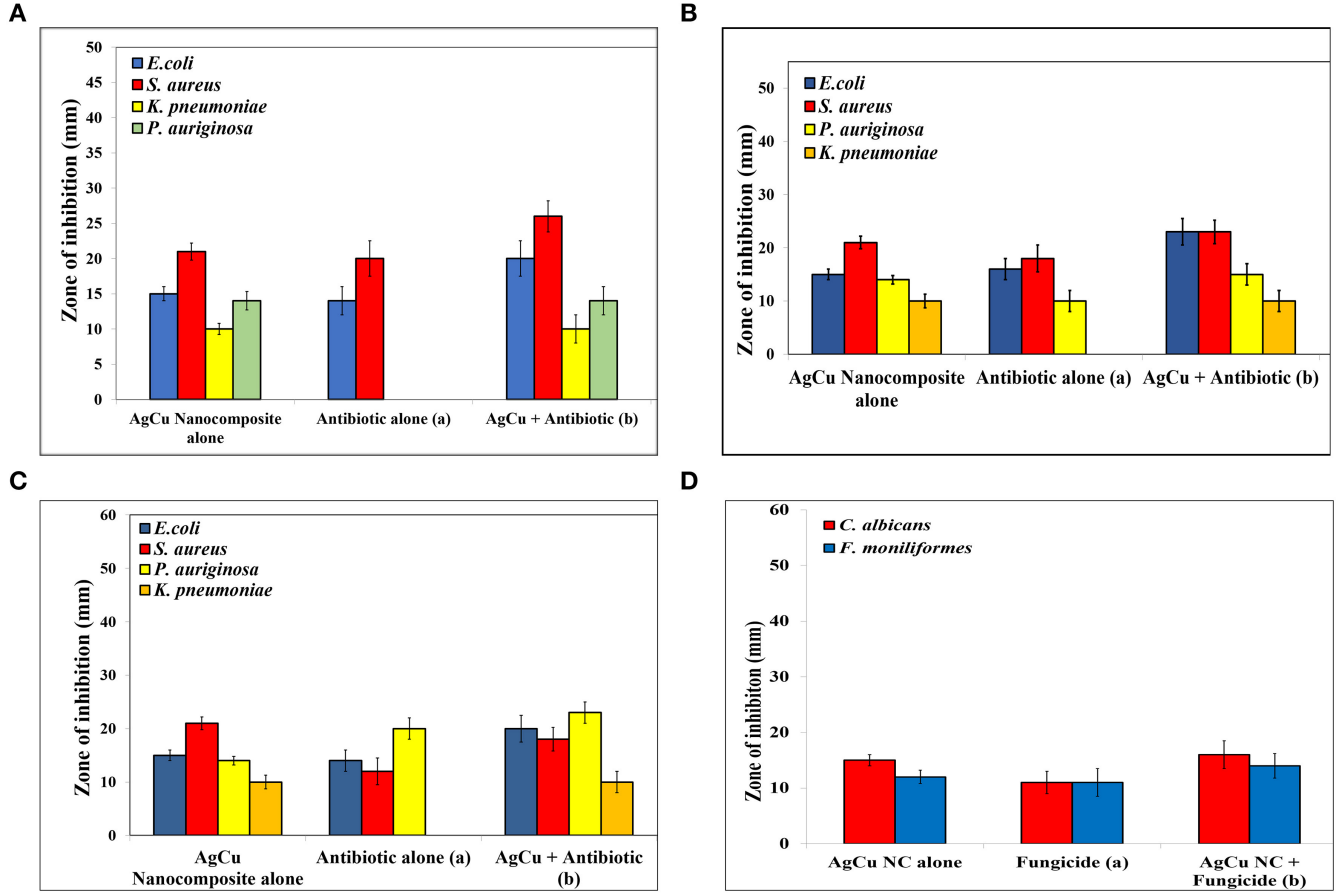
Antimicrobial potential of as-synthesized Ag-Cu NCs as expressed in terms of the zone of inhibition (in mm) in background of standard antibiotics **(A)** Ampicillin, **(B)** Kanamycin, **(C)** Gentamycin, and **(D)** Caspofungin (as control). As evident from the assay, Nanocomposites were found to be effective against antibiotic resistant strains as well. The combination therapy employing concomitant exposure with a combination of both nanocomposites and standard antibiotics increased the effectiveness of the blend by several folds.

The zone of inhibition assay further confirms the inhibitory effect of Ag-Cu NCs against the bacterial and fungal strains (Figure 4). The bactericidal effect of nanocomposites can be attributed to the combined antimicrobial effects possessed by silver and copper nanoparticles. The nanocomposites may reduce cell-to-cell interaction while internalization of nanocomposites in bacteria induces ROS production; in turn, this affects DNA and total cellular machinery, thereby killing microbial cells.

### Intracellular ROS Production by Ag-Cu Nanocomposites

**Figure 5:**
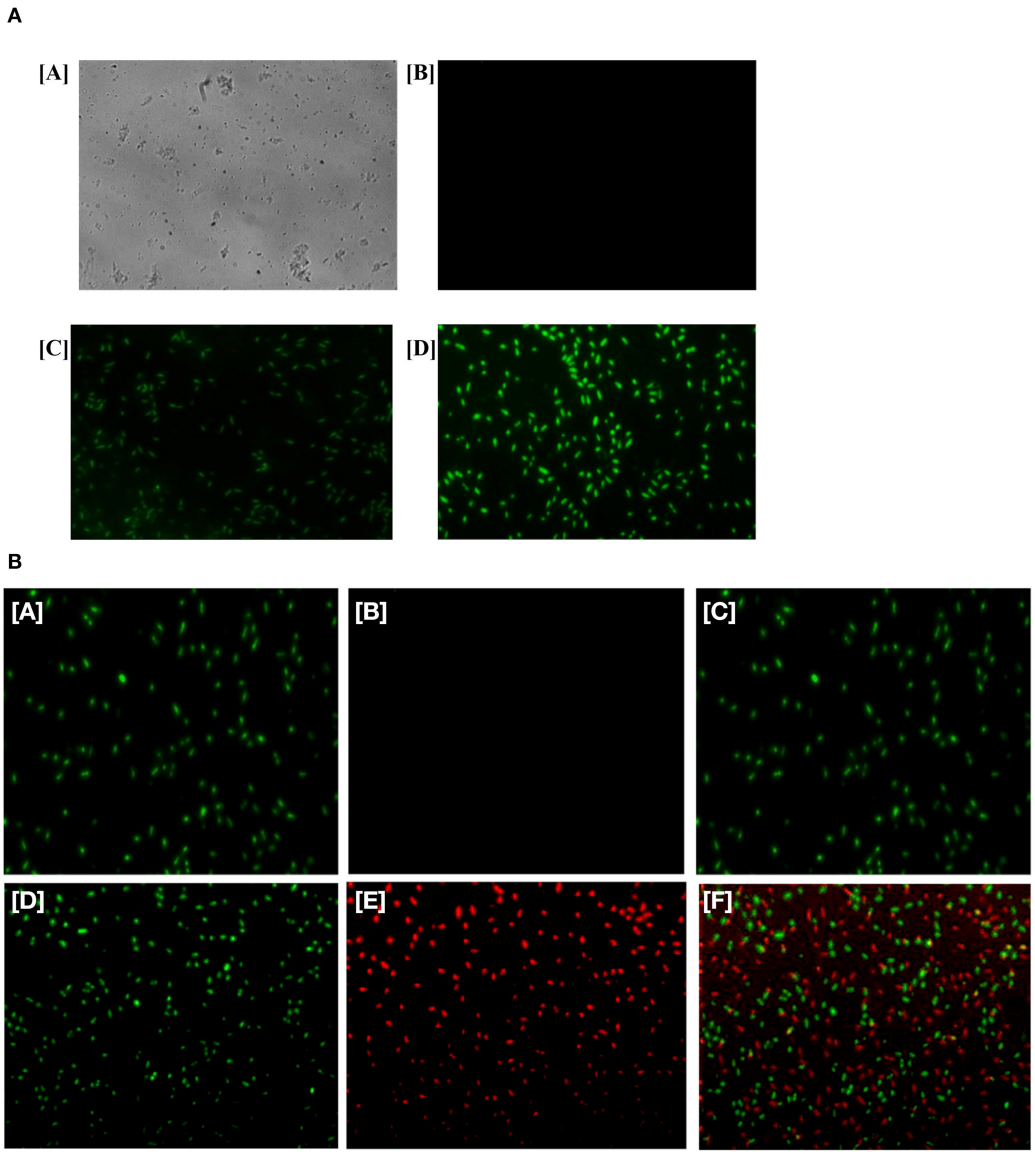
**(A)** Generation of ROS in the bacterial cells upon their treatment with as-formed Ag-Cu NCs. Micrograph showing [A] phase contrast picture of live cells, [B] fluorescence micrograph of untreated live cells, [C] micrograph depicting effect of as-generated ROS in the treated bacteria upon exposure to Ag-Cu NCs (62 μg/ml), and [D] an intensification in fluorescence upon treatment with increasing concentration of Ag-Cu NCs (125 μg/ml). **(B)** Fluorescence micrograph corresponding to live/dead assay employed to assess the antimicrobial activity of as-formed Ag-Cu NCs. (A, B, and C), untreated bacterial cells are stained with SYTO-9 only, as they fail to acquire PI fluorescence because of their intact membrane; (D, E, and F) micrographs correspond to bacterial cells post exposure to Ag-Cu NCs (125 μg/ml). The bacterial cells are stained with SYTO-9/PI post exposure to Ag-Cu NCs based formulation. The dead bacterial cells acquire PI fluorescence due to their broken membrane. The live cells acquire SYTO-9 stain [D], while dead bacteria are stained with PI [E]. The [F] panel corresponds to merge copy of [D, SYTO-9 fluorescence] and [E, PI fluorescence] images.

ROS production upon treatment of Ag-Cu NCs was assessed using the fluorescence dye, DCFH-DA (Figure 5A). In general, Ag-Cu NCs induces the generation of Reactive Oxygen Species (ROS) that causes alteration and decrementation of cellular proteins, DNA, and lipids, which can lead to cell death. In the presence of Reactive Oxygen Species, such as hydrogen peroxide and super oxide anion, DCFH-DA is oxidatively modified into a highly fluorescent derivative that is readily detectable under fluorescence microscope. As shown in Figure 5A, a significant increase in DCF fluorescence was observed upon treatment with Ag-Cu NCs.

### Live/Dead Assay to Ascertain Antimicrobial Potential of Various NCs/NPs

In order to establish the antimicrobial effect of Ag-Cu NCs against bacteria, we performed a live/dead assay employing Propidium Iodide (PI) dye. The cultured bacterial cells were treated with various nanoparticle-based formulations for 3 h. Subsequently, bacteria were stained with PI dye as described in the method section. PI penetrates the cells with damaged membrane (lesions). The dye PI binds with DNA to impart red color to the interacting bacteria. It should be noted that dye PI can access the DNA of only those cells that have a disrupted membrane. We found that PI dye had access to the cells that were damaged by Ag-Cu NCs. SYTO-9 stained only live cells and gave green fluorescence. The control group, which was not treated with the compound, failed to stain with PI (Figure 5B). The Ag-Cu NCs induced damage of target bacterial cells. This led to exposure of double stranded DNA of the cells. The PI dye can transverse damaged membrane of the treated cells and interact with the double stranded DNA. The fluorescence micrograph clearly indicated that the healthy control cells (untreated microbes) acquired the SYTO-9 dye color, while bacterial cells damaged by Ag-Cu NCs showed red fluorescence.

### Inhibition of Biofilm

**Figure 6:**
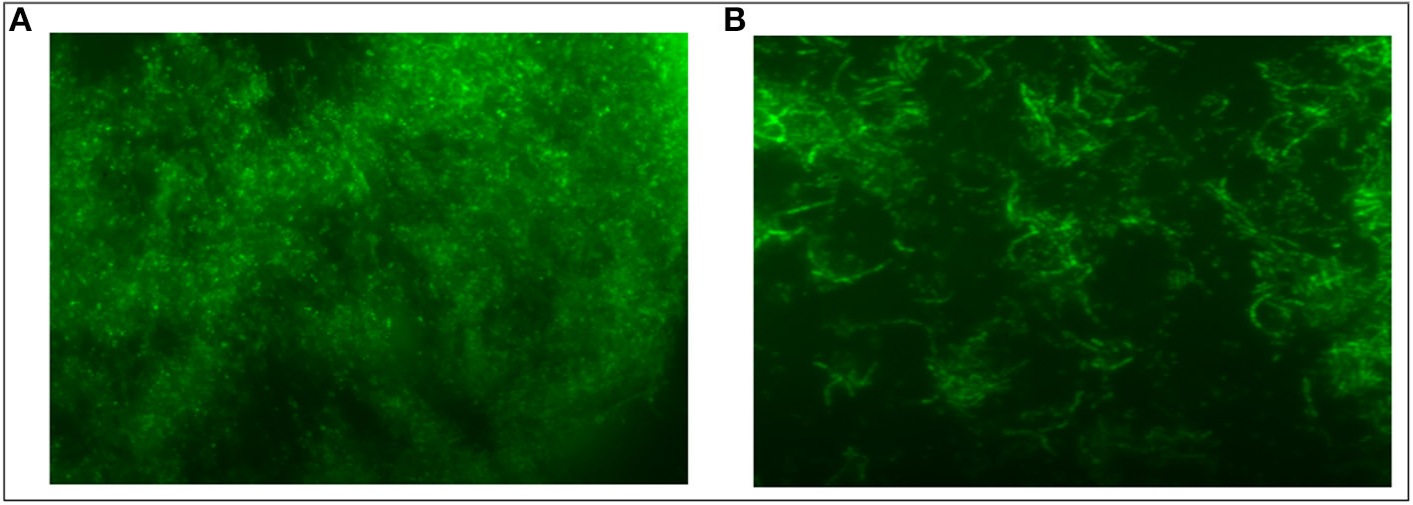
Inhibition of *S. aureus* Biofilm by Ag-Cu Nanocomposites: **(A)** biofilm formation by *S. aureus*, and **(B)** inhibition of biofilm in presence of as-synthesized Ag-Cu NCs (at their MIC value).

The biofilm inhibition was determined to assess the anti-biofilm potential of as-synthesized Ag-Cu NCs. The unhindered proliferation of untreated control cells resulted in the formation of profuse biofilm. On the other hand, the treatment with Ag-Cu NCs inhibited biofilm formation as depicted by in Figure 6.

## Discussion

Following advancement to the metallic nanoparticle era, the focus has now been shifted to the next-level nanocomposite-based hybrid system. Endowed with diversified attributes, the nanocomposites are much more promising compared to the monometallic nanoparticles. In the present study, we have synthesized silver-copper nanocomposite using an eco-friendly bio-mediated synthesis method. The reduction of metal salts and its subsequent capping (to prevent aggregation) was executed employing leaf extract of *Olax scandens*, a medicinally important plant. Besides facilitating bio-mediated synthesis of nanoparticles, *Olax* leaf extract has the unique property to impart fluorescence attributes to the as-synthesized particles. This opens a new vista into exploiting as-synthesized nanoparticles as a fluorescent probe to identify a range of target cells (Mujeeb et al., 2019). Moreover, the employed bio-mediated approach excludes the usage of harmful chemicals or high-temperature-based reducing conditions to fabricate nanoparticles, thus making the whole process more economic as well as ecofriendly (Tran and Le, 2013). The synthesis of Ag-Cu NCs was followed by UV-VIS spectrophotometric analysis. The UV-absorption spectrophotometric analysis of as-synthesized monometallic silver nanoparticles show λ-max at 480 nm, whereas monometallic copper nanoparticles show absorbance maxima at 560 nm. On the other hand, the as-synthesized nanocomposites were found out to absorb maximally at 520 nm, which is mid-way between the absorbance maxima of monometallic silver and copper nanoparticles. The kinetics of Ag-Cu NC synthesis suggests accomplishment of nanocomposite synthesis in 24 h. The concentration-dependent kinetic study suggests that the plant extract induced synthesis of as-synthesized NCs in concentration-dependent manner. This can be attributed to an increased amount of reducing and capping agent available (present in *Olax scandens* leaf extract) that helps in nanocomposite formation.

The fluorescence spectrum was recorded using an excitation wavelength at 320 nm. The emission spectrum was recorded in the range of 400–550 nm (in accordance with absorption spectra). The FTIR analysis showed the presence of various functional groups, such as carboxylate, alcoholic groups, amines, etc., that were present in both extracts and nanocomposites, thus suggesting that they were derived from plant extract. The size of the nanocomposites was in the range of 10–20 nm as revealed by TEM analysis. The unique physicochemical properties of the nanostructures with specific size distributed in a given range can be attributed to increased surface to mass ratio.

The antimicrobial potential of the nanocomposite was assessed against both Gram-negative and Gram-positive bacterial strains. We also determined anti-microbial potential of Ag-Cu NCs against some fungal isolates. The antimicrobial effect of plain as-synthesized silver and copper nanoparticles was compared with that of as-synthesized Ag-Cu NCs. We found that as-synthesized Ag-Cu NCs exhibited more potent anti-bacterial activity compared to plain NPs of the two metals. Agar disc diffusion assay further confirmed the inhibition of microbes as evident by clear zone of inhibition (Figure 4). Standard antibiotics were used as a control.

The efficacy of nanocomposites in combination with different antibiotics was also determined. A significant increase in the antimicrobial potential of antibiotics against various microbes was observed when a nanocomposite was supplemented with standard antibiotics. Interestingly, the combination showed remarkable antimicrobial potential against isolates of bacteria, which were not responding to antibiotic alone treatment. This suggests that as-synthesized Ag-Cu NCs can be used as an effective alternative antimicrobial agent against drug-resistant microbes as well. We tested antimicrobial potential of various as-synthesized NCs against both sensitive as well as resistant isolates of bacteria. In general, the bacterial isolates used in the present study were responding to several classes of antibiotics. However, some isolates showed resistance to one or other class of antibiotics. For example, among various bacterial isolates included in the study, *P. aeruginosa* did not respond to ampicillin alone. On the other hand, *K. pneumoniae* isolate did not respond to all three standard antibiotics viz ampicillin, kanamycin, and gentamycin. Interestingly, Ag-Cu NCs were found to be effective against all four types of clinical isolates including drug resistant isolates of *P. aeruginosa* and *K. pneumoniae*. The Ag-Cu NCs were effective against drug resistant isolates irrespective of their antimicrobial sensitivity to a given class of antibiotic. This suggests that Ag-Cu NCs employ an altogether different strategy (mechanism of bacterial killing) compared to the various antibiotics used in the present study for killing of target bacterial cells. The combination therapy employing supplementation of the standard antibiotics with Ag-Cu NCs also resulted in the successful killing of both sensitive as well as resistant isolates. Among the two as-formed plain metal-based NP formulations, AgNPs showed superior anti-microbial activity. The as-synthesized Ag-Cu NCs based formulation was found to effective against clinical stains of *K*. *pneumoniae* and *P. aeruginosa* isolates that were not responding to standard antibiotics, viz., ampicillin, kanamycin, and gentamycin. This is very incredible as more and more drug resistant isolates are emerging in Indian subcontinent and may pose great health hazards in near future.

**Figure 7:**
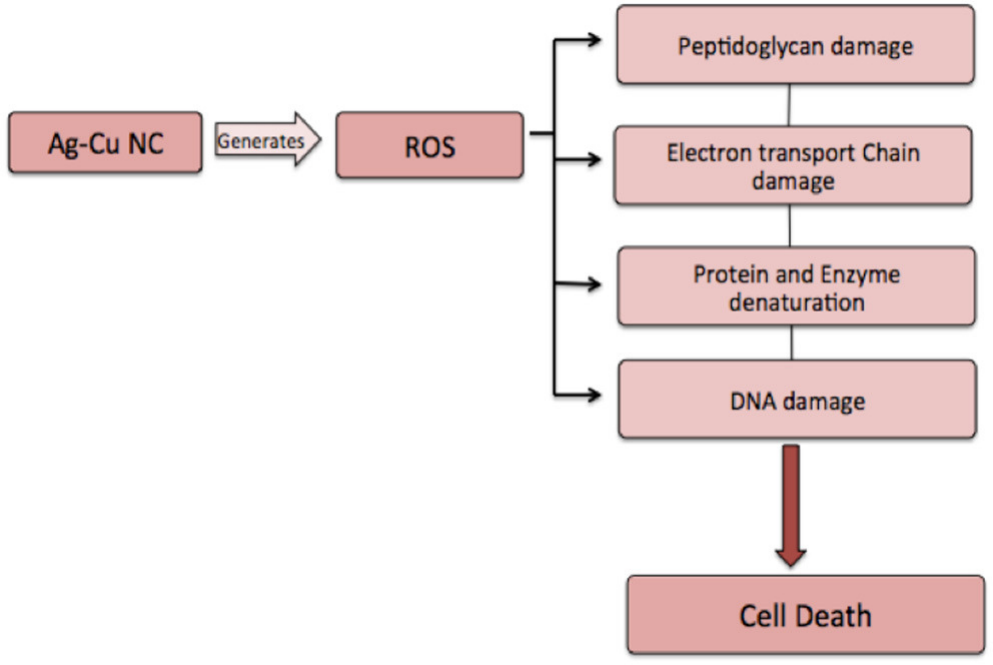
Proposed mechanism involved in anti-microbial action of Ag-Cu NCs. Ag-Cu NCs induced formation of ROS, which can damage the cell membrane and other cellular components, such as proteins, DNA, etc., thus leading to cell death.

After establishing antimicrobial potential against both sensitive as well as resistant isolates, we next tried to elucidate a possible operative mechanism responsible for observed antimicrobial activity (Figure 7). It was observed that Ag-Cu NCs induced ROS generation in the treated microbial cells (Figure 5A). The ROS produced by nanocomposites damaged the cellular proteins, DNA, and lipids of the treated cells and eventually lead to cell death. ROS generation was found to be directly proportional to the concentration of Ag-Cu NCs. A Live/Dead assay using PI and SYTO-9 dye further confirmed the anti-microbial potential of as-synthesized Ag-Cu NCs (Figure 5B).

Biofilms are comprised of microbial cells that are embedded in a self-synthesized matrix consisting of extracellular polymeric substance (EPS) formed by polysaccharides, proteins, lipids, and extracellular DNA (e-DNA) etc. Bacterial cells in a biofilm become a thousand times more resistant to antibiotics as compared to the free forms. Various mechanisms, involved at both the cellular as well as the community level, contribute to the microbial film-dependent antibiotic resistance of microbial cells. These mechanisms may involve the development of enzymatic resistance as well as the chemical modification to the target domains of the antibiotic. Biofilm provides a protective layer to the constituent cells because it limits the penetration of the antibiotic into the cells. Interestingly, as-synthesized Ag-Cu NCs also demonstrated strong anti-biofilm activity against *S. aureus* biofilm.

The proposed bio mediated synthesis of Ag-Cu NCs can be a promising approach for efficient, low-cost, and non-toxic upscale production of nanocomposites. This may find tremendous scope and application in pharmaceutical application. The effectiveness of silver-copper nanocomposites against drug-resistant bacterial strains makes it a promising substitute to the existing antibiotics to fight resistance menace. Interestingly, the ability of a composite to induce ROS may find an application in the killing of cancer cells as well. The concomitant anti-cancerous and anti-microbial activity along with fluorescent attributes will make reported as-synthesized NCs a potent theranostic approach with great biomedical applications.

## Data Availability Statement

All datasets generated for this study are included in the article.

## Author Contributions

AM and MO conceived and designed the experiments. AM, NK, KB, FJ, AA, and HS performed the experiments. AM, NK, MK, and SK analyzed the data. MO and IG contributed the reagents, materials, and analysis tools. AM wrote the first draft of the manuscript.

### Conflict of Interest

The authors declare that the research was conducted in the absence of any commercial or financial relationships that could be construed as a potential conflict of interest.
