# Corrigendum: *Olax scandens* Mediated Biogenic Synthesis of Ag-Cu Nanocomposites: Potential Against Inhibition of Drug-Resistant Microbes

**DOI:** 10.3389/fchem.2020.00822

**Published:** 2020-09-16

**Authors:** Anzar Abdul Mujeeb, Nuha Abeer Khan, Fauzia Jamal, Khan Farheen Badre Alam, Haris Saeed, Shadab Kazmi, Ansam Wadia Faid Alshameri, Mohammad Kashif, Irfan Ghazi, Mohammad Owais

**Affiliations:** ^1^Interdisciplinary Biotechnology Unit, Aligarh Muslim University, Aligarh, India; ^2^Plant Molecular Biology and Genetic Engineering Division, The National Botanical Research Institute, Council of Scientific and Industrial Research, Lucknow, India; ^3^Department of Biochemistry, School of Life Sciences, University of Hyderabad, Hyderabad, India

**Keywords:** biogenic, Ag-Cu NCs, anti-biofilm potential, reactive oxygen species (ROS), antimicrobial potential

In the original article, there was a mistake in Figure 5A as published. An incorrect image was used in panel C. The corrected Figure 5A appears below.

There was also a mistake in the legend for Figure 5 as published. The correct legend appears below.

**FIGURE 5:**
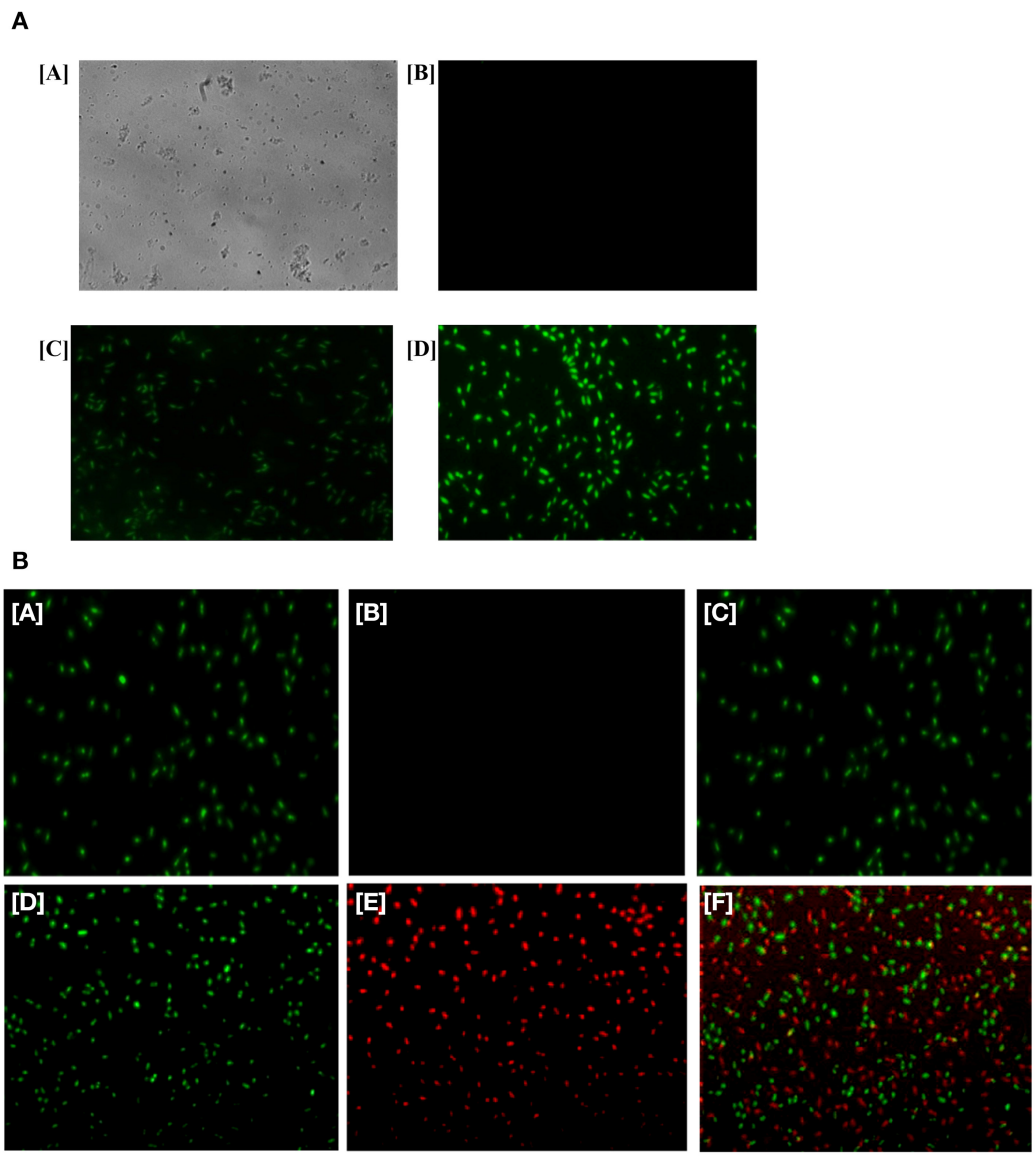
**(A)** Generation of ROS in the bacterial cells upon their treatment with as-formed Ag-Cu NCs. Micrograph showing [A] phase contrast picture of live cells, [B] fluorescence micrograph of untreated live cells, [C] micrograph depicting effect of as-generated ROS in the treated bacteria upon exposure to Ag-Cu NCs (62 μg/ml), and [D] an intensification in fluorescence upon treatment with increasing concentration of Ag-Cu NCs (125 μg/ml). **(B)** Fluorescence micrograph corresponding to live/dead assay employed to assess the antimicrobial activity of as-formed Ag-Cu NCs. (A, B, and C), untreated bacterial cells are stained with SYTO-9 only, as they fail to acquire PI fluorescence because of their intact membrane; (D, E, and F) micrographs correspond to bacterial cells post exposure to Ag-Cu NCs (125 μg/ml). The bacterial cells are stained with SYTO-9/PI post exposure to Ag-Cu NCs based formulation. The dead bacterial cells acquire PI fluorescence due to their broken membrane. The live cells acquire SYTO-9 stain [D], while dead bacteria are stained with PI [E]. The [F] panel corresponds to merge copy of [D, SYTO-9 fluorescence] and [E, PI fluorescence] images.

The authors apologize for these errors and state that they do not change the scientific conclusions of the article in any way. The original article has been updated.

